# Mesenchymal Stem Cells in Restoration of Fertility at Experimental Pelvic Inflammatory Disease

**DOI:** 10.1155/2017/2014132

**Published:** 2017-08-27

**Authors:** Nataliia Volkova, Mariia Yukhta, Anatoliy Goltsev

**Affiliations:** Department of Cryopathophysiology and Immunology, Institute for Problems of Cryobiology and Сryomedicine of the National Academy of Sciences of Ukraine, Pereyaslavskaya Str. 23, Kharkov 61015, Ukraine

## Abstract

Inflammatory disorders account for a significant percentage of gynecologic diseases, particularly in women of reproductive age. It is known that stem cells have anti-inflammatory and regenerative properties. Based on this, we investigated the effect of intravenous administration of cryopreserved mesenchymal stem cells (cMSCs) of bone marrow on experimental chronic inflammation of the ovaries. The paper shows that on the 21st day after cMSC therapy, leukocyte infiltration of ovaries was slightly relative to the control group without treatment, and the ratio of developing and atretic follicles in the animals with cMSC injection dramatically increased, while in the control, it still remained on the side of atretic forms. The number of apoptotic oocytes after stimulation of superovulation in the control group was significantly higher (85.3 ± 5.2%) than that in the animals with therapy (5.7 ± 0.8%). Relative number of fertilized eggs in the group with cMSC therapy was higher by 40% compare to that in the control. Pregnancy rate in natural estrous cycle after cell administration increased by 20%, and average number of litters in this group was two times significantly higher than that in the control. So the intravenous injection of cMSCs has the restorative effect on the fertility at experimental pelvic inflammatory disease.

## 1. Introduction

Chronic pelvic inflammatory disease associated with lower abdominal pain, low fever, irregular menstruation, and dysmenorrhea, is a leading cause of infertility and ectopic pregnancy and is related to an increased risk of ovarian borderline tumor. Pelvic inflammatory disease typically occurs as microorganisms ascend from the lower genital tract, infecting the uterus, fallopian tubes, and ovaries [1]. The microbial etiology is linked to sexually transmitted microorganisms, including *Chlamydia trachomatis, Neisseria gonorrhoeae, Mycoplasma genitalium*, and bacterial vaginosis-associated microorganisms, predominantly anaerobes [1]. Patients with chronic pelvic inflammatory disease often suffer from acute attack or immune deficiency, and inappropriate antibiotic management may result in endometritis, oophoritis, tubo-ovarian abscess, or peritonitis [2].

This pathology for many years has remained one of the most currency problems in clinical practice due to the high rate, despite the advances in prevention, diagnostic, and treatment [3]. It has a negative impact on the reproducibility of women, their ability to give birth to a healthy child [4]. Practical gynecologists are facing a number of difficulties in treatment of this pathology. Currently, up to 80% of pelvic inflammatory disease is manifested in chronic forms that are often hardly treated with standard protocols [5].

It is known that chronic inflammation has an adverse effect on ovarian reserve. In a study [6], a lower level of anti-Müllerian hormone that is a sensitive indicator for ovarian reserve was observed in women with bilateral tube occlusion compared with normal tubes. Besides, changes in index of blood flow in the ovary and uterine tube have been observed in rats with aseptic inflammation [7]. Decreased blood flow, in turn, increases the incidence of apoptotic granulosa cells and leads to the dysfunction in steroidogenesis synthesis [8]. Moreover, it can also lead to chronic inflammation accompanied with possible excessive DNA damages [9, 10]. Since oocytes are very vulnerable to ubiquitous external damage, the number of damaged follicles associated with chronic inflammation may increase, leading to a reduction of ovarian reserve through atresia [11].

The analysis of publications shows the relevance of investigations aimed to improve the reproductive function in animals and humans using cell therapy. The bone marrow transplantation restored oocyte production in wild-type mice sterilized by chemotherapy, as well as in ataxia telangiectasia-mutated gene-deficient mice, which are otherwise incapable of making oocytes [12].

In a study [13], authors demonstrated that grafted human amnion epithelial cells could migrate into injured ovarian tissue and differentiated into granulosa cells around oocytes. It was shown that the mRNA levels of proinflammatory cytokines (such as TNF-*α*, IL-8, and IL-1*β*) are significantly upregulated, and their elevation was observed in conjunction with the increase in follicular atresia in the ovaries of chemoablated mice.

There is an evidence of positive impact of mesenchymal stem cells (MSCs) on the treatment of gynecological diseases [14–17] too. In addition to their stem/progenitor properties, MSCs have also been shown to possess broad immunoregulatory abilities and are capable of influencing both adaptive and innate immune responses. Recent findings have demonstrated that MSCs actively interact with components of the innate immune system; through that, they display anti-inflammatory effect [18–21].

The homing ability of MSCs is significant therapeutically because it allows their easy administration [22, 23]. The cells can be administrated intravenously, and after homing to the sites of inflammation, they will perform local immunoregulatory actions.

Modern technologies of culture and cryopreservation make possible the receiving of stem cells reserve with subsequent long-term storage at low temperatures without significant changes in functional state. This allows a free transport of cells and their thawing just before therapeutic use.

The aim of this study was to investigate the effect of intravenous administration of cryopreserved mesenchymal stem cells (cMSCs) of bone marrow on experimental chronic inflammation of the ovaries.

## 2. Materials and Methods

### 2.1. Experimental Animals

Eight-week-old female white mice (*n* = 70, weighing 18–20 g) were used in the experiment. The animals were housed in plastic cages (one animal per cage) and kept at the controlled temperature (18–22°C), humidity (30%–70%), and lighting (lights on from 8 a.m. to 8 p.m.) on a standard diet with free access to food and water. Mice were acclimated for at least seven days prior to experiments. During housing, animals were monitored daily for health status. No adverse events were observed.

In addition, seven white mice (weighing 20–25 g) were used for isolation of MSCs.

All the manipulations were carried out in a strict accordance with the requirements of the “European Convention for the Protection of Vertebrate Animals used for Experimental and other Scientific Purposes”. Animals were sacrificed by CO_2_ asphyxiation in all series of experiment. The protocol was approved by the Committee in the Bioethics of Animal Experiments of the Institute for Problems of Cryobiology and Сryomedicine of the National Academy of Sciences of Ukraine (Permit Number 2014-02).

### 2.2. Isolation, Culture, and Cryopreservation of MSCs

MSCs were isolated from resected femur of mice by washing out with Hanks' solution (PAA, Pasching, Austria) followed by flushing through needles with gradually decreased diameter. The next step was centrifugation at 834 ×g for 5 min. The cells were resuspended in culture medium and plated on culture flasks (PAA) with 10^3^ cells per cm^2^ density. Cultural medium contained Iscove's Modified Dulbecco's Medium (PAA), 10% fetal bovine serum (FBS) (HyClone, Logan, UT, USA), gentamicin (150 mg/ml) (Farmak, Kiev, Ukraine), and amphotericin B (10 mg/ml) (PAA). Cultural medium was changed every three days. We used standard culture conditions (37°C, 5% CO_2_, and 95% humidity) in a CO_2_ incubator (Sanyo, Osaka, Japan). MSCs were detached at 80% confluence with 0.25% trypsin-EDTA (Hyclone) and were replanted in other flasks with 1 : 2 ratios. Third passage of MSCs was used in all experiments.

Cryopreservation solution was the growth medium supplemented with 10% DMSO (PanEco, Moskow, Russia) and 20% FBS. Freezing was performed at 1°C/min to −80°C followed by plunging into liquid nitrogen [24]. All samples were stored in a low-temperature bank for three months and thawed in a water bath at 40°C to a liquid phase. Cryoprotectant was removed by slowly adding a 10-fold volume of Hanks' solution (PAA) followed by centrifugation at 834 ×g for 5 min. After being thawed, cells were immediately used for therapy. After freeze/thawing, the immune phenotype and apoptotic/necrotic processes in cells were investigated with FACS Calibur using antirat primary monoclonal antibodies raised against CD34, CD44, CD73, CD105, and annexin V with 7-amino-actinomycin D (7AAD) (BD and US), respectively. The results were analyzed with WinMDI v.2.8 software. The proliferative characteristics of MSCs were examined by MTT test (Sigma, US) on the 1st, 3rd, 7th, and 10th days of culture [25].

### 2.3. Oophoritis Simulation in Mice

**Figure 1:**
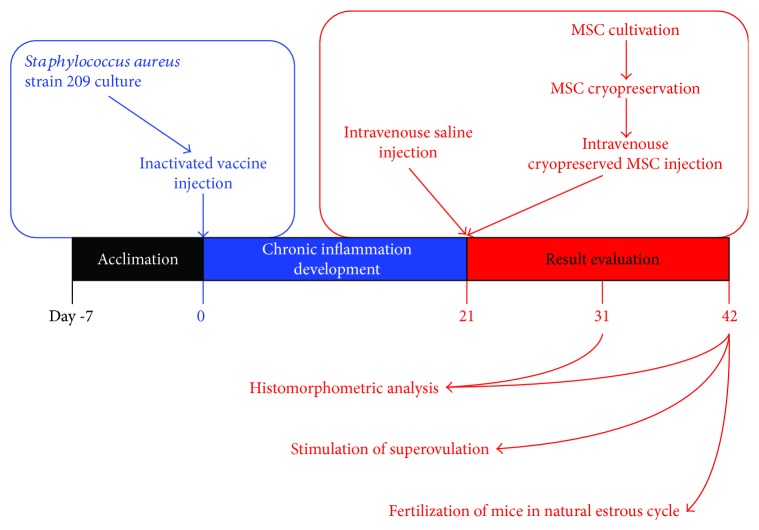
Experimental schedule. Mice were acclimated for at least 7 days prior to experiments. Then, they were intraperitoneally injected with inactivated vaccine of *Staphylococcus aureus* to simulate the chronic inflammation of ovaries. 21 days later, control animals were intravenously injected with saline solution and experimental ones were intravenously injected with cMSCs. The results were evaluated on the 10th and 21st days after therapy (31st and 42nd days of experiment).

In order to simulate the chronic inflammation of ovaries, the mice were intraperitoneally injected with inactivated vaccine of *Staphylococcus aureus* strain 209 (50 × 10^6^ microbial bodies in 0.3 ml of saline). Then, the animals were kept under aforementioned conditions during 21 days without treatment (Figure 1).

To obtain the inactivated vaccine, a daily *S. aureus* culture was prepared, washed off from agar with 5 ml of saline, and incubated at 75°C for one hour. After incubation, the suspension was diluted to the desired concentration and used in the experiment.

### 2.4. Cell Therapy

On the 22nd day from the moment of inactivated vaccine administration, the animals were randomly divided in two groups. The mice in the control group (*n* = 25) were intravenously injected with saline solution (0.2 ml). The mice in the experimental group (*n* = 25) were intravenously injected with cMSCs (0.5 × 10^6^ of viable cells in 0.2 ml of Hanks' solution). In addition, twenty animals of corresponding age and weight were used in the experiment as intact control.

### 2.5. Histomorphometric Analysis

For histological study, the ovaries (*n* = 5 from each group of animals) were cut off with surrounding strands of omentum on the 10th and 21st days after cell administration and fixed in 10% aqueous neutral formalin solution and then the serial paraffin sections with 4-5 *μ*m thickness were done and stained with hematoxylin and eosin. The sections were examined and photographed with the light microscope (Carl Zeiss, Oberkochen, Germany).

The development stages of follicles were identified as reported [26]. According to this system, primordial follicle is determined as dormant and small with only one layer of granulosa cells. Primary follicle is determined as oocyte surrounded by a single layer of cubic granulosa cells. Preantral follicle has an oocyte, surrounded by multiple layers of granulosa cells which isolate from theca cells by the basement membrane. If the follicle is increased in volume and oocyte in it is surrounded by several layers of granulosa cells forming a cavity that contains follicular fluid, it is considered as antral one.

Follicles from right and left ovaries were counted, and their average number per section was used for statistical analyses. Due to the average diameter of a primordial oocyte is 8 p.m., they were counted in every 10th section, and the result was multiplied by 5 to determine the actual number per ovary. All other classes of follicles were counted in every section with the nucleolus of the oocyte used as a marker. The follicles were counted as atretic if the oocyte exhibited evidence of degeneration such as eosinophilic inclusions, meiotic resumption, nuclear clumping, or oocyte atrophy.

### 2.6. Superovulation in Mice

On the 21st day after the beginning of the therapy, a superovulation was induced in mice according to the next procedure: they were intramuscularly injected with 5 IU of pregnant mare serum gonadotropin (“Intervet,” Netherlands) and with 7.5 IU of human chorionic gonadotropin in a 46–48 hr interval (“Organon,” Netherlands) [27].

The oocytes were collected from the prepared oviducts of animals (*n* = 5 from each group of animals) 12-13 hrs after the injection of human chorionic gonadotropin. Cumulus cells were removed using 1% hyaluronidase solution (+37°C). Presence/absence of apoptosis in the oocytes was assessed with annexin V. Staining was performed according to the standard procedure of the manufacturer.

For determining the relative number of fertilized eggs after stimulation of superovulation, the females (*n* = 5 from each group of animals) were placed to the fertile males just after the injection of human chorionic gonadotropin for a night. The fact of mating was identified by a copulative plug next morning. The two-cell embryos (if fertilization has occurred) or oocytes (if fertilization has not occurred) were collected from the prepared oviducts not earlier than 48 hrs after the last injection and counted.

### 2.7. Fertilization of Mice in Natural Estrous Cycle

For fertilization in natural estrous cycle, each female (*n* = 5 from each group of animals) on the 21st day from the beginning of therapy was placed to a fertile male for 5 days (1 estrous cycle). The pregnancy rate was calculated as a ratio of females which became pregnant to the total number of animals in the group multiplied by 100%. After birth of litters, their average number per one female, survival rate (the ratio of mice survived up to the 5th day to those which were living at birth), and dynamics of body weight during the first 15 days of life were determined.

### 2.8. Statistical Analysis

A single-factor analysis of variance and the Student *t*-test were used for statistical processing of the results with the software “Statistica 8.” The critical level of significance equaled to 0.05. Results are presented as the means and standard errors (M ± SE).

## 3. Results

### 3.1. Сharacteristics of Cryopreserved MSCs before Administration

The cytofluorimetric analysis showed that cryopreservation did not cause the development of apoptosis/necrosis in MSCs (Table 1).

The percentage of CD44 positive cells in MSC and cMSC suspensions was 96.21 ± 0.22% and 92.53 ± 0.24%; CD73 was 94.14 ± 0.12% and 96.98 ± 0.22%; CD105 was 97.16 ± 0.25% and 94.32 ± 0.21%, respectively. Furthermore, MSCs and cMSCs were negative for the hematopoietic lineage marker CD34, indicating that they had nonhematopoietic origin.

**Figure 2:**
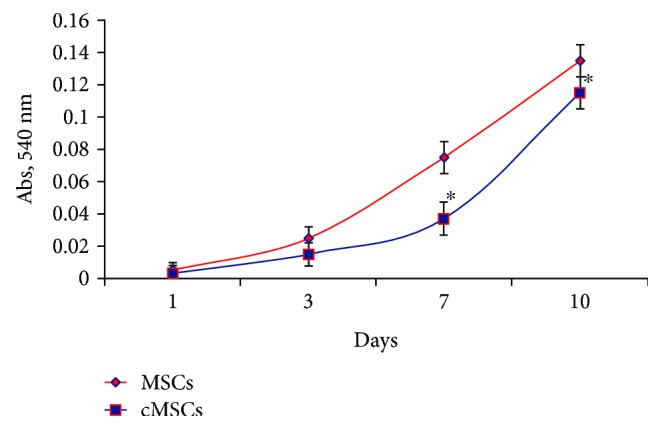
Proliferation of MSCs after cryopreservation. On the first passage, cMSCs were characterized by a lower proliferation rate, and on the 10th day, they reached only 65% of confluent versus 80% density of monolayer in cultures without cryopreservation. ^∗^Statistically significant difference relatively to MSCs at the same term of cultivation (*p* < 0.05).

In studied cultures, the adhered cells had fibroblast-like morphology and formed the monolayer sites with 10–15 cells on the 2nd-3rd days of cultivation. On the 10th day, bone marrow MSCs reached 80% of confluent, and in the case of cMSCs, the density of monolayer was 65% (Figure 2). It should be noted that on the 3rd-4th passages, the investigated cryopreserved cultures had higher ability to proliferation versus passage 0.

### 3.2. Histological Evaluation of the Ovaries

**Figure 3:**
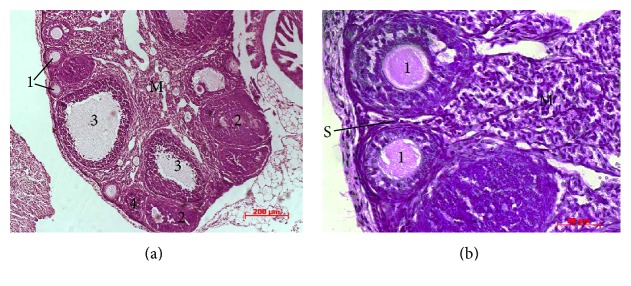
Histological structure of intact ovaries. Ovarian follicles in different stages of development (primary (1), preantral (2), and antral (3) follicles) and corpora lutea (4) were separated by a stroma (S). The environment of antral follicles is homogeneous with poor eosin staining. The medulla of ovaries (M) consisted mainly of loose connective tissue and blood vessels. Hematoxylin and eosin staining.

Histological examinations showed that the ovaries of intact animals had a normal histological structure (Figure 3). A large number of ovarian follicles in different stages of development (primordial, primary, preantral, and antral follicles) and corpora lutea with radial strands of luteum cells were separated by a stroma. The environment of antral follicles was homogeneous with poor eosin staining; oocytes in them were evenly surrounded with granulosa cells. Single atretic follicles with typical histological structure were defined on the periphery of sections. The medulla of ovaries consisted mainly of loose connective tissue and blood vessels.

In our previous studies, it was shown that intraperitoneal injection of inactivated vaccine of *Staphylococcus aureus* strain 209 on the 21st day led to the formation of chronic productive inflammation in mice ovaries, which accompanied by dominance of atresia over the follicle growth with the development of sclerotic changes and their total number reducing [27].

**Figure 4:**
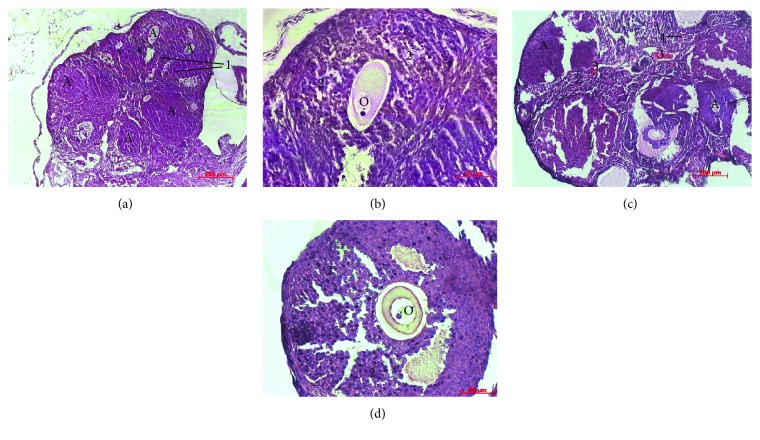
Histological sections of the ovaries on the 10th day of experiment. (a), (b) After saline administration: Ovarian follicle atresia (A) was occurred by productive type and characterized by deformed contour (1) and formation of multilayer capsule from granulosa cells (2), which were compressed, separated, and were less adjacent to oocyte (O). The layers of granulosa cells. (c), (d) After cMSC administration: The number of atretic follicles and severity of productive processes in them decreased compare to the control and the full-blooded capillaries (3), and hemorrhages (4) were determined in the stroma. Hematoxylin and eosin staining.

In the animals from control group on the 10th day of observation, the leukocyte infiltration was persisted in all the layers of ovaries. The number of developing follicles in the cortex was reduced, and most of the remaining follicles were visibly degenerating (Figures 4(a) and 4(b)). Ovarian follicle atresia was occurred by productive type and characterized by formation of multilayer capsule from granulosa cells with deformed contour; the layers of which were compressed, separated, and were less adjacent to oocyte. It is known that disturbance of contact between oocyte and granulosa cells is irreversible and affects negatively the oocyte metabolism and development [28]. There was no functionally active corpora lutea, and the interstitium was hypertrophic. At the same time, the morphological structure of ovaries on the 10th day after cMSC injection had a positive dynamics of development (Figures 4(c) and 4(d)). Intensity of leukocyte infiltration in ovaries was significantly lower compared to that in the control group. The number of atretic follicles and severity of productive processes in them decreased too. Follicular profile in this group of animals was presented by primordial, primary, and preantral follicles.

**Figure 5:**
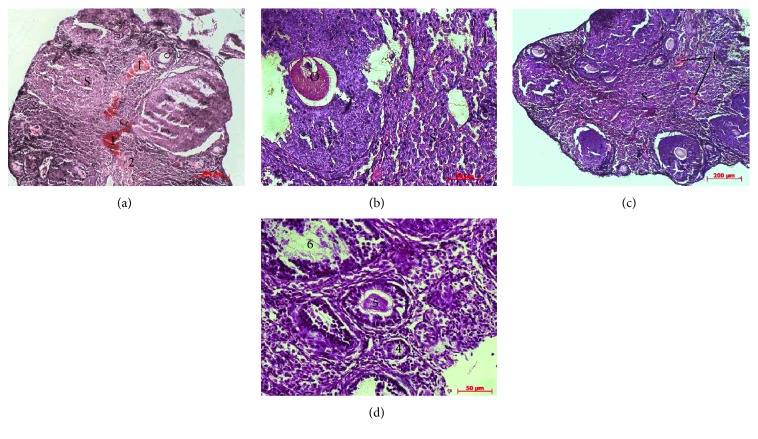
Histological sections of the ovaries on the 21st day of experiment. (a), (b) After saline administration: Primordial and primary follicles were not available; the extensive area of ovaries was filled with stroma (S) in which the full-blooded capillaries (1) and hemorrhages (2) were determined. Granulosa cells (3) were compressed, separated, and were less adjacent to oocyte (O). (c), (d) After cMSC administration: The follicular profile was characterized by the presence of primordial (4), primary (5), and preantral (6) follicles. Hematoxylin and eosin staining.

With extended observation period up to the 21st day, further degradation of the ovarian structural components occurred in the control group (Figures 5(a) and 5(b)). Thus, an intense leukocyte infiltration was observed over the entire thickness of ovaries. Primordial and primary follicles in most cases were not available, and the detecting ones had the signs of degeneration. Preantral and antral follicles were not found in any cases. The extensive area of ovaries was filled with stroma in which the full-blooded capillaries and hemorrhages were determined.

Meanwhile, in the group with cMSC therapy at this term of observation, a tendency to normalization of ovary morphological parameters was observed (Figures 5(c) and 5(d)). Slight leukocyte infiltration was observed only in the cortical layer that pointed to the reduction of inflammation intensity relatively to the control group and previous term of observation. The follicular profile was characterized by the presence of primordial, primary, and preantral follicles and in some cases, by the single antral follicles.

Morphometric study demonstrated that the average follicular number per one ovary on the 10th day had statistically significant reduction (*p* < 0.05) both in the control and experimental groups compared with that in the intact animals (Table 2).

**Figure 6:**
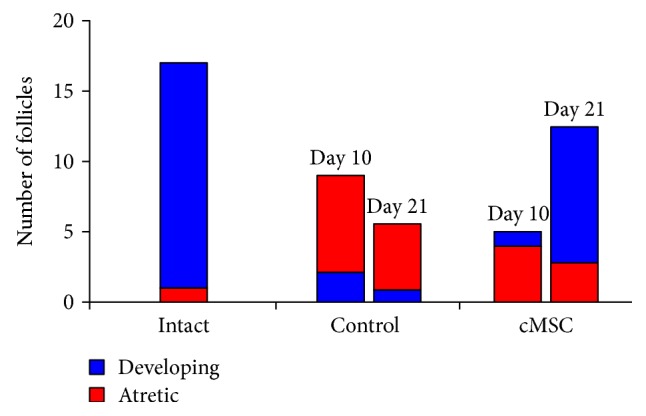
The number of developing and atretic follicles in the ovaries. In the control group, atretic follicles strongly dominated over developing forms on the 10th day, and after cell administration, their number was almost equal. On the 21st day, the ratio of developing and atretic follicles in the ovaries of animals with cMSC injection dramatically increased, while in the control, it still remained on the side of atretic ones.

It should be noted that in the control group, atretic follicles strongly dominated over developing forms, and after cell administration, their number was almost equal (Figure 6). On the 21st day in animals with cMSC therapy, the average follicular number increased in comparison to the control group without statistically significant difference with values of the intact animals (Table 2). The ratio of developing and atretic follicles in the ovaries of animals with cMSC injection dramatically increased, while in the control, it still remained on the side of atretic forms (Figure 6).

### 3.3. Stimulation of Superovulation

In the series of experiments to study the quality of oocytes and their ability to fertilization after oophoritis therapy, we obtained the results which showed that the number of apoptotic oocytes after stimulation of superovulation in the control group was 85.3 ± 5.2%, and in the animals with cMSC injection, it was significantly lower (5.7 ± 0.8%), while in intact animals, this parameter was equal to 1.2 ± 0.3%. The relative number of fertilized eggs, developed up to the two-cell embryos, after fertilization of intact females was 98.8 ± 0.7%. In the control group, this index did not exceed 56.5 ± 5.5%. The number of embryos in the group with cMSC therapy was higher by 36% (77.9 ± 6.3%) compared to that in control animals.

### 3.4. Fertilization in Natural Estrous Cycle

The pregnancy rate of intact animals was 86.6 ± 5.4%. Under a chronic inflammation of the ovaries and injection of saline, a pregnancy rate in natural estrous cycle decreased 1.6 times if compared to that in the intact animals (Table 3). Cell therapy contributed to the increase of this index by 20% if compared with the control but it did not reach the level of intact animals.

### 3.5. Postnatal Development

The average number of litters in offspring of intact animals was within physiological values (Table 3). In the control group, this value was 2.2 times lower compared to those in the intact mice. In the group with cMSC therapy, the average number of litters was significantly higher by 98% than that in the control.

Postnatal development of offspring within the period from birth to the 5th day of life in all groups of animals was characterized by the high survival rate: for intact animals, this index made 97.4 ± 1.2%, for the control and experimental groups, it was 96.5 ± 1.1% and 95.9% ± 0.9%, respectively.

**Figure 7:**
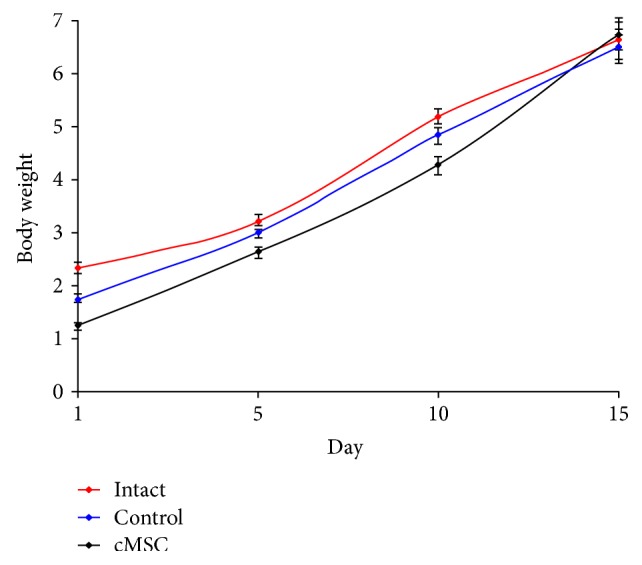
Dynamics of body weight of litters. There was a slow rise of body weight in the control group relative to ones from other groups at early observation terms, and to the 15th day, this difference leveled. ^∗^Statistically significant difference with corresponding index in the intact group (*p* < 0.05).

Body weight dynamics of litters during the first 10 days of life was slightly different between the groups (Figure 7).

There was a slow increase of body weight in the control group relatively to the animals from the intact and experimental ones at early observation terms. But to the 15th day, this difference leveled.

## 4. Discussion

We evaluated the effects of cMSC transplantation on fertility in mice with chronic ovarian inflammation. It was shown that the intravenous injection of cMSC produced a modulatory effect on the course of inflammation and had a restorative effect on the fertility suggesting that this procedure may be useful in patients with chronic pelvic inflammatory disease. The transplantation of cMSCs resulted in the restoration of folliculogenesis, improvement of the oocytes' quality, and increase of the relative number of embryos obtained after stimulation of superovulation as well as the pregnancy rate and average number of litters in natural estrous cycle, compared with animals that did not receive cell therapy.

Thus, our findings demonstrated that on the 21st day after cMSC therapy, leukocyte infiltration reduced relative to the control group, and follicular profile was characterized by the presence of primordial, primary, preantral, and single antral follicles that pointed on follicular reserve restoration. The determination of number of apoptotic oocytes after stimulation of superovulation showed that in the control group, this index was significantly higher relative to the group of animals with cell therapy. Therapeutic effect of MSCs was also manifested in increase of relative number of fertilized eggs, developed up to the two-cell embryos by 40% compared to the control. Along with this, pregnancy rate in natural estrous cycle after cell therapy increased by 20%, and average number of litters in this group was two times significantly higher than that in the control.

Corrective effect of intravenous injection of bone marrow cMSCs on the restoration of fertility in mice is likely associated with anti-inflammatory and regenerative properties. One of the mechanisms behind this is the integration of MSCs into the tissue and replacement of damaged cells; the other one is the paracrine mediators secreted by MSC which also might be involved in the repair by inducing angiogenesis, preventing cell apoptosis, and promoting functional recovery [16, 29]. Whether or not mammalian females generate new oocytes during adulthood from germline stem cells to sustain the ovarian follicle pool has recently generated controversy [30].

In 2004, Johnson et al. [31] reported surprising findings that germline stem cells are capable to generate oocytes in the adult mouse ovaries. In 2005, the same research group based on the findings of germline markers in bone marrow declared that mammalian oocytes originate from putative germ cells in bone marrow and are distributed by peripheral blood to the ovaries [12]. Later, a number of scientists also announced the existence of germline stem cells [32–34]. But this data seems to be controversial. Thus, Reize et al. [35] showed that in the reconstructed mouse cell lineage trees, oocytes form clusters that are separate from hematopoietic and mesenchymal stem cells, both in young and old mice, indicating that these populations belong to distinct lineages. Another scientist also showed that adult female mice neither require nor contain active germline stem cells or produce new oocytes in vivo [30]. Nevertheless, it is not excluded that MSCs can migrate into injured ovarian tissue and also differentiated into granulosa cells around oocytes as it has been shown for human amniotic epithelial cells [13, 36].

The cytokines secreted by MSCs may also play an important supportive role in the ovarian microenvironment. Fu et al. [16] have demonstrated that MSC secretes significant amounts of VEGF, IGF-1, and HGF in vitro, through which they may participate in granulosa cell and follicular growth. It is known that VEGF is an angiogenic cytokine that promotes the proliferation of endothelial cells and formation of new vessels. It also reduces rat ovarian granulosa cell damage by inhibiting apoptosis [37]. IGF-1 also increased granulosa cell proliferation, decreased apoptosis, and promoted follicular antrum formation [38, 39]. Furthermore, IGF-1 amplify gonadotropin hormone action in terms of increased steroidogenesis by ovarian granulosa cell and granulosa cell proliferation [40]. Recent studies have shown that HGF can suppress apoptosis in ovarian granulosa cells and mediates the positive feedback loop between theca cells and granulosa cells, which can promote the dramatic cell growth required for folliculogenesis [41, 42].

Taken together with our findings described in this study, it appears that the possible mechanisms by which cMSCs participate in ovary repair are the inducing angiogenesis, increasing the follicular growth and improving the quality of oocytes and their ability to fertilization due to paracrine activity of administrated cells. So, the intravenous injection of cMSCs is safe and noninvasive procedure, and therefore, it can be a perspective method in the development of complex therapy of women with chronic oophoritis and associated infertility.

## 5. Conclusions

Thus, an intravenous injection of cryopreserved bone marrow-derived mesenchymal stem cells to the animals with chronic oophoritis contributes to the restoration of ovarian reserve. They produce a modulatory effect on the course of inflammation, restore folliculogenesis, improve the quality of oocytes, and increase the relative number of embryos obtained after stimulation of superovulation as well as the pregnancy rate and average number of litters in natural estrous cycle.

## Figures and Tables

**Table 1:** Cytofluorimetric analysis of MSCs after thawing and staining with annexin V and 7AAD.

Group	Аnnexin V^+^/7AAD^−^	Аnnexin V^−^/7AAD^−^	Аnnexin V^+^/7AAD^+^ + annexin V^−^/7AAD^+^
MSCs	3.87 ± 2.63	84.08 ± 2.91	12.05 ± 1.22
cMSCs	4.69 ± 2.16	76.16 ± 9.61	19.15 ± 7.24

**Table 2:** The average number of follicles in the ovaries.

Group	Day of the therapy	
	10	21
Control group (saline injection)	10.6 ± 2.48^#^	7.4 ± 2.18^#^
Experimental group (cMSC injection)	9.4 ± 2.63^#^	15.3 ± 1.80^∗^
Intact animals	18.25 ± 4.52	

^∗^Statistically significant difference with corresponding index in the control group (*p* < 0.05); ^#^statistically significant difference with corresponding index of intact animals (*p* < 0.05).

**Table 3:** The pregnancy rate and number of litters in offspring of females fertilized in natural estrous cycle.

Group	Pregnancy rate (%)	Number of litters
Control group (saline injection)	53 ± 8.9^#^	4.2 ± 1.3^#^
Experimental group (cMSC injection)	73 ± 6.9^∗^^,#^	8.3 ± 1.8^∗^
Intact animals	86 ± 5.4^∗^	9.4 ± 2.4^∗^

^∗^Statistically significant difference with corresponding index in the control group (*p* < 0.05); ^#^statistically significant difference with corresponding index of intact animals (*p* < 0.05).

## References

[1] Soper D. E. (2010). Pelvic inflammatory disease. *Obstetrics and Gynecology*.

[2] Rasmussen C. B., Faber M. T., Jensen A. (2013). Pelvic inflammatory disease and risk of invasive ovarian cancer and ovarian borderline tumor. *Cancer Causes & Control*.

[3] Scholes D., Stergachis A., Heidrich F. E., Andrilla H., Holmes K. K., Stamm W. E. (1996). Prevention of pelvic inflammatory disease by screening for cervical chlamydial infection. *The New England Journal of Medicine*.

[4] Brunham R. C., Gottlieb S. L., Paavonen J. (2015). Pelvic inflammatory disease. *The New England Journal of Medicine*.

[5] Mitchel C., Prabhu M. (2013). Pelvic inflammatory disease: current concepts in pathogenesis, diagnosis and treatment. *Infectious Disease Clinics of North America*.

[6] Cui L., Sheng Y., Sun M., Hu J., Qin Y., Chen Z.-J. (2016). Chronic pelvic inflammation diminished ovarian reserve as indicated by serum anti-Mülerrian hormone. *PLoS One*.

[7] Borodin I. I., Ustiugov E. D., Sklianova N. A., Liubarskii M. S. (1991). The morphometric characteristics of the blood microcirculatory bed of the ovary and uterine tube in rats with aseptic inflammation and after the use of a carbon-mineral sorbent. *Arkhiv Anatomii, Gistologii I Embriologii*.

[8] Du B., Takahashi K., Ishida G. M., Nakahara K., Saito H., Kurachi H. (2006). Usefulness of intraovarian artery pulsatility and resistance indices measurement on the day of follicle aspiration for the assessment of oocyte quality. *Fertility and Sterility*.

[9] Lin R., Xiao D., Guo Y. (2015). Chronic inflammation-related DNA damage response: a driving force of gastric cardia carcinogenesis. *Oncotarget*.

[10] Kiraly O., Gong G., Olipitz W., Muthupalani S., Engelward B. P. (2015). Inflammation-induced cell proliferation potentiates DNA damage-induced mutations in vivo. *PLoS Genetics*.

[11] Roos W. P., Kaina B. (2013). DNA damage-induced cell death: from specific DNA lesions to the DNA damage response and apoptosis. *Cancer Letters*.

[12] Johnson J., Bagley J., Skaznik-Wikiel M. (2005). Oocyte generation in adult mammalian ovaries by putative germ cells in bone marrow and peripheral blood. *Cell*.

[13] Qiuwan Z., Minhua X., Xiaofen Y., Ting L., Qian W., Dongmei L. (2015). Human amniotic epithelial cells inhibit granulosa cell apoptosis induced by chemotherapy and restore the fertility. *Stem Cell Research & Therapy*.

[14] Komarova S., Roth J., Alvarez R., Curiel D. T., Pereboeva L. (2010). Targeting of mesenchymal stem cells to ovarian tumors via an artificial receptor. *Journal of Ovarian Research*.

[15] Takehara Y., Yabuuchi A., Ezoe K. (2013). The restorative effects of adipose-derived mesenchymal stem cells on damaged ovarian function. *Laboratory Investigation*.

[16] Fu X., He Y., Xie C., Liu W. (2008). Bone marrow mesenchymal stem cell transplantation improves ovarian function and structure in rats with chemotherapy-induced ovarian damage. *Cytotherapy*.

[17] Li W. N., Xie Q. X., Qin J. W. (2011). Effect of mesenchymal stem cell transplantation on immunological injury of the ovary in mice. *Journal of Southern Medical University*.

[18] Prockop D. J., Oh J. Y. (2012). Mesenchymal stem/stromal cells (MSCs): role as guardians of inflammation. *Molecular Therapy*.

[19] Bernardo M. E., Fibbe W. E. (2013). Mesenchymal stromal cells: sensors and switchers of inflammation. *Cell Stem Cell*.

[20] Wang L., Zhao Y., Shi S. (2012). Interplay between mesenchymal stem cells and lymphocytes. Implications for immunotherapy and tissue regeneration. *Journal of Dental Research*.

[21] Gao F., Chiu S. M., Motan D. A. L. (2016). Mesenchymal stem cells and immunomodulation: current status and future prospects. *Cell Death & Disease*.

[22] Becker A., Riet I. V. (2016). Homing and migration of mesenchymal stromal cells: how to improve the efficacy of cell therapy?. *World Journal of Stem Cells*.

[23] Abhishek S., Verfaillie C. M. (2013). Mesenchymal stem cells migration homing and tracking. *Stem Cells International*.

[24] Volkova N. A., Goltsev A. N. (2015). Сryopreservation effect on proliferation and differentiation potential of cultured chorion cells. *CryoLetters*.

[25] Mossman T. (1983). Rapid colorimetric assay for cellular growth and survival: application to proliferation and cytotoxicity assays. *Journal of Immunological Methods*.

[26] Myers M., Britt K. L., Wreford N. G., Ebling F. J., Kerr J. B. (2004). Methods for quantifying follicular numbers within the mouse ovary. *Reproduction*.

[27] Monk M. (1987). *Mammalian Development: A Practical Approach*.

[28] Gu L., Liu H., Gu X., Boots C., Moley K. H., Wang Q. (2015). Metabolic control of oocyte development: linking maternal nutrition and reproductive outcomes. *Cellular and Molecular Life Sciences*.

[29] Xu M., Uemura R., Dai Y., Wang Y., Pasha Z., Ashraf M. (2007). In vitro and in vivo effects of bone marrow stem cells on cardiac structure and function. *Journal of Molecular and Cellular Cardiology*.

[30] Lei L., Spradling A. C. (2013). Female mice lack adult germ-line stem cells but sustain oogenesis using stable primordial follicles. *Proceedings of the National Academy of Sciences of the United States of America*.

[31] Johnson J., Canning J., Kaneko T., Pru J. K., Tilly J. L. (2004). Germline stem cells and follicular renewal in the postnatal mammalian ovary. *Nature*.

[32] Zou K., Yuan Z., Yang Z. (2009). Production of offspring from a germline stem cell line derived from neonatal ovaries. *Nature Cell Biology*.

[33] White Y. A., Woods D. C., Takai Y., Ishihara O., Seki H., Tilly J. L. (2012). Oocyte formation by mitotically active germ cells purified from ovaries of reproductive-age women. *Nature Medicine*.

[34] Pacchiarotti J., Maki C., Ramos T. (2010). Differentiation potential of germ line stem cells derived from the postnatal mouse ovary. *Differentiation*.

[35] Reize Y., Itzkovitz S., Adar R. (2012). Cell lineage analysis of the mammalian female germline. *PLoS Gene*.

[36] Wang F., Wang L., Yao X., Lai D., Guo L. (2013). Human amniotic epithelial cells can differentiate into granulosa cells and restore folliculogenesis in a mouse model of chemotherapy-induced premature ovarian failure. *Stem Cell Research and Therapy*.

[37] Shin S. Y., Lee J. Y., Lee E. (2006). Protective effect of vascular endothelial growth factor (vegf) in frozen-thawed granulosa cells is mediated by inhibition of apoptosis. *European Journal of Obstetrics Gynecology and Reproductive Biology*.

[38] Mao J., Smith M. F., Rucker E. B. (2004). Effect of epidermal growth factor and insulin-like growth factor I on porcine preantral follicular growth, antrum formation, and stimulation of granulosal cell proliferation and suppression of apoptosis in vitro. *Journal of Animal Science*.

[39] Sirotkin A. V., Dukesova J., Pivko J., Kubek A. (2002). Effect of growth factors on proliferation, apoptosis and protein kinase a expression in cultured porcine cumulus oophorus cells. *Reproduction Nutrition Development*.

[40] Behl R., Kaul R. (2002). Insulin like growth factor 1 and regulation of ovarian function in mammals. *Indian Journal of Experimental Biology*.

[41] Uzumcu M., Pan Z., Chu Y., Zachow R. (2006). Immunolocalization of the hepatocyte growth factor (hgf) system in the rat ovary and the anti-apoptotic effect of hgf in rat ovarian granulosa cells in vitro. *Reproduction*.

[42] Ito M., Harada T., Tanikawa M., Fujii A., Shiota G., Terakawa N. (2001). Hepatocyte growth factor and stem cell factor involvement in paracrine interplays of theca and granulosa cells in the human ovary. *Fertility and Sterility*.

